# Peptide of *Trichinella spiralis* Infective Larval Extract That Harnesses Growth of Human Hepatoma Cells

**DOI:** 10.3389/fcimb.2022.882608

**Published:** 2022-04-26

**Authors:** Pichet Ruenchit, Onrapak Reamtong, Ladawan Khowawisetsut, Poom Adisakwattana, Monrat Chulanetra, Kasem Kulkeaw, Wanpen Chaicumpa

**Affiliations:** ^1^ Department of Parasitology, Faculty of Medicine Siriraj Hospital, Mahidol University, Bangkok, Thailand; ^2^ Department of Molecular Tropical Medicine and Genetics, Faculty of Tropical Medicine, Mahidol University, Bangkok, Thailand; ^3^ Department of Helminthology, Faculty of Tropical Medicine, Mahidol University, Bangkok, Thailand; ^4^ Center of Research Excellence in Therapeutic Proteins and Antibody Engineering, Department of Parasitology, Faculty of Medicine Siriraj Hospital, Mahidol University, Bangkok, Thailand

**Keywords:** antitumor peptide, drug discovery, human hepatocellular carcinoma HepG2 cell, infective larva, proteomics, *Trichinella spiralis*

## Abstract

*Trichinella spiralis*, a tissue-dwelling helminth, causes human trichinellosis through ingestion of undercooked meat containing the parasite’s infective larvae. However, benefits from *T. spiralis* infection have been documented: reduction of allergic diseases, inhibition of collagen-induced arthritis, delay of type 1 diabetes progression, and suppression of cancer cell proliferation. Since conventional cancer treatments have limited and unreliable efficacies with adverse side effects, novel adjunctive therapeutic agents and strategies are needed to enhance the overall treatment outcomes. This study aimed to validate the antitumor activity of *T. spiralis* infective larval extract (LE) and extricate the parasite-derived antitumor peptide. Extracts of *T. spiralis* infective larvae harvested from striated muscles of infected mice were prepared and tested for antitumor activity against three types of carcinoma cells: hepatocellular carcinoma HepG2, ovarian cancer SK-OV-3, and lung adenocarcinoma A549. The results showed that LE exerted the greatest antitumor effect on HepG2 cells. Proteomic analysis of the LE revealed 270 proteins. They were classified as cellular components, proteins involved in metabolic processes, and proteins with diverse biological functions. STRING analysis showed that most LE proteins were interconnected and played pivotal roles in various metabolic processes. *In silico* analysis of anticancer peptides identified three candidates. Antitumor peptide 2 matched the hypothetical protein T01_4238 of *T. spiralis* and showed a dose-dependent anti-HepG2 effect, not by causing apoptosis or necrosis but by inducing ROS accumulation, leading to inhibition of cell proliferation. The data indicate the potential application of LE-derived antitumor peptide as a complementary agent for human hepatoma treatment.

## Introduction

*Trichinella spiralis* is a nematode that infects humans, carnivores, and omnivores, and causes trichinellosis (also known as trichinosis) (Despommier, 1993). Reservoir hosts of this parasite are pigs, wild boars, horses, and wildlife (Murrell, 2013; Hidalgo et al., 2019; Rawla and Sharma, 2020). The parasite life cycle comprises three stages. The first is infective muscle larvae (ML) that encyst in nurse cells formed in striated muscles of the infecting host. The predilection sites are functionally active muscles, *e.g*., the diaphragm, intercostal, lingual, laryngeal, masseter, and ocular muscles. The second stage is newborn larvae (NL), while the third is adult male and female worms (AW) (Despommier, 1993). Humans are infected through the consumption of undercooked meat containing ML. After ingestion, ML develop into AW in the small intestine, where mating occurs, followed by NL production. The NL are released into circulation, spread throughout the host tissues, and finally form infective larvae that thrive in the muscle nurse cells (Sofronic-Milosavljevic et al., 2015). Clinical manifestations of human trichinellosis vary from generalized fever, diarrhea, abdominal pain, nausea, vomiting, and myalgia to more severe ailments, such as difficulty with movement coordination, labored breathing, myocarditis, and encephalitis (Rawla and Sharma, 2020).

In contrast to the deleterious effects on infected humans, *T. spiralis* infection and parasite products are helpful in medical applications for different maladies. *Trichinella spiralis* infection delays the onset and modulates the progression of type 1 diabetes (Saunders et al., 2007). Excretory‐secretory products (ESP) of *T. spiralis* ML have anti-inflammatory activity. Specifically, a 45-kDa glycoprotein showed an inhibitory effect on neutrophil inflammatory function (Bruschi et al., 2000). In addition, ML-ESP reduced the progression and severity of experimental autoimmune encephalomyelitis (EAE) in mice and modulated human dendritic cell activation (Kuijk et al., 2012). Extracellular vesicles of the *T. spiralis* ML carry immunomodulatory proteins that were shown to increase the production of anti-inflammatory myokine, interleukin 6 (IL–6), and immunosuppressive cytokine, IL–10 (Kosanović et al., 2019).

Furthermore, recombinant 53-kDa protein of the parasite protected mice against colitis (Du et al., 2011) and acute lung injury caused by bacterial lipopolysaccharide by alleviating lung pyroptosis through the promotion of M2 macrophage polarization (Wei et al., 2021). A mixture of crude extracts from *T. spiralis* AW and NL had antineoplastic activity against human erythroleukemic K562 and H7402 hepatoma cells (Wang et al., 2009). Human hepatoma H7402 cells transfected with the recombinant pEGFP-N1-A200711 gene of *T. spiralis* underwent apoptosis; thus, the protein encoded by the A200711 gene was proposed as a therapeutic agent for hepatocellular carcinoma (Wang et al., 2013). The excretory-secretory product of *T. spiralis* ML showed inhibitory activity against human lung adenocarcinoma A549 cells and induced tumor cell cycle arrest and apoptosis *via* the mitochondrial pathway (Wu et al., 2020). Moreover, *T. spiralis* proteins may inhibit sarcoma (Lubiniecki and Cypess, 1975; Molinari et al., 1979), melanoma (Kang et al., 2013), myeloma (Gong et al., 2011), stomach cancer (Wang et al., 2009), and breast cancer (Kristek et al., 2005). However, details on the *T. spiralis* proteins/peptides that manifest anticancer activity are limited.

In this study, we extended the antitumor activity of the *T. spiralis* ML extract to hepatic, ovarian, and lung cancers. Proteins and peptides of the ML extract that caused detrimental effects to human hepatoma cells were investigated, using proteomic and bioinformatic guidance and experimental validation.

## Materials and Methods

### Animal Ethics and Biosafety

The animal husbandry and experiments were reviewed and approved by the Mahidol University-Institute Animal Care and Use Committee (MU-IACUC) of the Faculty of Medicine Siriraj Hospital, Mahidol University (project code, SI-ACUP 007/2562; approval number 014/2562) and the Siriraj Safety Risk Management Taskforce, Mahidol University (approval number SI 2019-013). Animal manipulation was performed by scientists and veterinarians holding certificates for experimental animal care and use certified by the National Research Council of Thailand.

### Cancer Cells and Cultures

Cells from human hepatocellular carcinoma (HepG2), ovarian cancer (SK-OV-3), and human adenocarcinomic alveolar basal epithelium (A549) were grown in T 25-cm^2^ flasks (Corning, Steuben County, NY, USA) in Dulbecco’s modified Eagle’s medium (DMEM) supplemented with 10% heat-inactivated fetal bovine serum (FBS; HyClone, GE Healthcare Bio-Sciences Austria GmbH, Pasching, Austria) and antibiotics (100 UmL^-1^ penicillin and 100 µgmL^-1^ streptomycin; Gibco, Life Technologies Corporation, NY, USA; complete DMEM) at 37°C in a humidified 5% CO_2_ atmosphere. Trypsin-EDTA (0.05%) was used to detach the cells from the culture flasks.

### Preparation of *T. spiralis* Infective Larval Extract

BALB/cAJcl mice infected with infective muscle larvae (ML) of *T. spiralis* for 90 days were euthanized by intraperitoneal injection of an overdose of thiopentone (150 mg per kg body weight). Encysted *T. spiralis* larvae were isolated from the skeletal muscles by the digestion method (Crum et al., 1977) but with slight modification. Briefly, the deskinned and eviscerated carcasses were minced in a meat grinder and digested with pepsin-HCl solution (0.8% [w/v] pepsin and 1% [v/v] hydrochloric acid in normal saline solution [NSS]) at 37°C for 1 h. Muscle larvae were isolated by passing the digested material through five layers of gauze. The larvae retained on the gauze were flushed by washing. They were allowed to set by gravitation in a conical glass jar, washed twice with NSS, sedimented by centrifugation (200 × *g*, 4 °C, 5 min), and resuspended in sterile phosphate-buffered saline (PBS; pH 7.4).

The larval suspension was treated with a 0.05% (v/v) protease inhibitor cocktail (PIC; Set III, Merck, Darmstadt, Germany). They were then ground with a plastic pestle and sonicated at 20 kHz on ice for 5 min (Omni-Ruptor 4000, OMNI-INC, Kennesaw, GA, USA). The larval homogenate was centrifuged (10 000 × *g*, 4 °C, 10 min), the supernatant (infective larval extract; LE) was collected, and the protein content was determined (BCA assay, SMART BCA Protein Assay Kit, Intronbio, South Korea).

### Determination of LE Antitumor Activity by Tumor Cell Proliferation Assay

The antitumor activity of LE was evaluated *in vitro* against the three tumor cell lines (HepG2, SK-OV-3, and A549) using the CellTiter 96 Non-Radioactive Cell Proliferation Assay (Promega, Madison, WI, USA). Briefly, aliquots of individual cancer cells (5 000 cells in 100 µL of complete DMEM) were seeded into separate wells of 96-well culture plates (triplicate) and incubated at 37°C in a CO_2_ incubator for 24 h. The culture fluids were discarded, and the cells were replenished with 100 µL of fresh culture medium containing *T. spiralis* LE (70 µgmL^-1^) and incubated for a further 24 h. Medium supplemented with 0.05% (v/v) PIC was used as a negative control. Growth inhibition was evaluated and expressed as percentage cell survival. Three independent experiments were performed, with results presented as mean ± standard deviation (SD). The inhibitory concentration 50 (IC_50_) value of the LE was calculated using probit analysis with data of percentage cell survival of the tumor cell that was least tolerant to LE treatment (Postelnicu, 2011). Morphological changes in the treated cells were observed microscopically (Olympus IX70 Inverted Tissue Culture Microscope, Olympus, Tokyo, Japan) at 100× magnification and recorded by a WiFi Microscope Digital Camera (Model MC4KW-G1, Microscope X, JiangSu, China).

### Proteomic Analysis of *T. spiralis* Infective LEs

Proteins in the LE were studied through gel-based proteomic analysis using electrospray ionization quadrupole time-of-flight mass spectrometry (ESI-QUAD-TOF). Briefly, proteins in the LE were subjected to 12% sodium dodecyl sulfate-polyacrylamide gel electrophoresis (SDS-PAGE), stained with Coomassie Brilliant Blue R-250 dye (CBB), and digested by trypsin (Phumisantiphong et al., 2017). The generated peptides were subjected to an Ultimate 3000 nano-LC system (Dionex, Surrey, UK). The eluted peptides were directly analyzed by a MicroToF Q II mass spectrometer (Bruker, Bremen, Germany). The LC-MS/MS raw data files were processed and converted into the Mascot Generic File (.mgf) format using DataAnalysis software, version 3.4. The.mgf files were searched using Mascot version 2.3.02 (Matrix Science, London, UK) against the NCBI database. *Trichinella spiralis* was set for the taxonomy filter. Missed cleavages were set to 1. Peptide tolerance and tandem MS tolerance were set to 0.8 Da and 0.8 Da, respectively. The variable modification was set to carbamidomethylation and oxidation.

All identified proteins of the *T. spiralis* LEs were (1) studied using the BLAST top-hit species distribution, (2) classified based on the cellular component, and (3) categorized according to their functions in the biological process and molecular functions, using the Blast2GO tool of OmicsBox software (Götz et al., 2008). Moreover, the protein-protein interaction network was analyzed using Search Tool for the Retrieval of Interacting Genes (STRING; http://string-db.org) online database (Szklarczyk et al., 2019). The minimum required interaction score was defined as medium confidence (> 0.4) and medium FDR stringency (5%).

### *In silico* Analysis of *T. spiralis* Peptides With Antitumor Activity

To explore the antitumor peptide candidates, predictions were made of the antitumor activities of 44 peptides from the proteomic results that hit the *T. spiralis* proteins. Two bioinformatic tools were used: AntiCP (Agrawal et al., 2021) and ACPred-FL (Wei et al., 2018); both are web-based, anticancer peptide prediction servers. The support vector machine (SVM) score and the number of positive and negative motifs were recorded after analysis by the AntiCP algorithm. The prediction result with the percentage of confidence was recorded after analysis by the ACPred-FL algorithm. Selected candidates were predicted for peptide structure using PEP-FOLD3 (Lamiable et al., 2016). The results are shown as a ribbon diagram with a local structure prediction profile.

### Evaluation of Antitumor Activity of the Peptide Candidates

Based on the *in silico* analysis, peptides with antitumor potential were chosen. The peptides were synthesized commercially (GenScript, Piscataway, NJ, USA) and tested for their antitumor activity as described above. HepG2 cells (5 × 10^3^ cells per well) were treated with different concentrations (5.5, 11, 22, 44, 88, 176, and 352 µgmL^-1^) of each peptide candidate. Cells treated with medium alone and medium supplemented with 1% dimethyl sulfoxide (DMSO) were negative and diluent controls, respectively. The inhibitory activity of each peptide candidate was studied after 24 h of treatment using a CellTiter 96 Non-Radioactive Cell Proliferation Assay (Promega). The inhibitory concentration 50 (IC_50_) value was calculated using probit analysis. The viable cell number and morphological changes of the treated cells were observed microscopically at 200× magnification using an Olympus IX70 inverted tissue culture microscope (Olympus). Three independent experiments were performed; results are shown as mean ± standard deviation (SD).

### Determination of Peptide-Mediated Cancer Cell Apoptosis by Flow Cytometry

To investigate whether the antitumor peptide inhibits HepG2 cell proliferation *via* apoptosis, HepG2 cells (5 × 10^5^ cells in 6-well-culture plate) were treated with 337 µgmL^-1^ of selected antitumor peptide and controls, *i.e*., 1% DMSO (diluent control), medium alone (negative control), and 50 µM of camptothecin (apoptosis mediated control), for 24 h. After treatment, cells were harvested, washed once with PBS, and once with 1× binding buffer (eBioscience Annexin V Apoptosis Detection Kit FITC, Thermo Fisher Scientific, Waltham, MA, USA). Cell pellets were resuspended in 100 µL of 1× binding buffer containing 5 µL of fluorochrome-conjugated Annexin V and 3 µL of propidium iodide (PI) and kept at room temperature (25 °C) in darkness for 15 min. After that, samples were mixed with 400 µL of 1× binding buffer and immediately analyzed by BD LSRFortessa Cell Analyzer (BD Bioscience, San Jose, CA, USA) using 485 nm excitation and 535 nm emission wavelengths.

### Determination of Cellular Oxidative Stress

Oxidative stress in HepG2 cells was determined using a DCFDA/H2DCFDA-Cellular Reactive Oxygen Species (ROS) Assay Kit and fluorescence microscopy. HepG2 cells (1 × 10^5^ cells on chamber slides in 24-well culture plates) were treated with 337 µgmL^-1^ of the antitumor peptide of interest, 50 µM of tert-butyl hydrogen peroxide (tbHP; positive oxidant control), 1% DMSO (diluent control), and medium alone (negative control) for 24 h. The generation of ROS in the treated HepG2 cells was then determined using the DCFDA/H2DCFDA-Cellular ROS Assay Kit (Abcam, Cambridge, MA, USA). The treated cells were washed with 1× buffer, stained with 2’, 7’-dichlorofluorescin diacetate (DCFDA) solution (1× buffer containing 10 µM of DCFDA), and incubated at 37 °C for 45 min in darkness, as per the manufacturer’s instructions. After incubation, the stained cells were washed twice with 1× buffer, fixed with cold methanol at room temperature for 20 min, and placed on a glass slide containing 8 µL of mounting medium. Cellular ROS production was observed under confocal microscopy (Nikon Instruments Inc., Melville, NY, USA) at 400× magnification with a filter set appropriate for fluorescein (FITC). Intensity mean value of FITC signal was analyzed using Zen 3.4 (blue edition) software (Zeiss, Oberkochen, Germany).

### Statistical Analyses

Data were analyzed using IBM SPSS Statistics for Windows, version 21 (IBM Corp., Armonk, NY, USA). One-way ANOVA followed by the Tukey HSD *post hoc* test for multiple comparisons were used to compare the mean difference between each group of treatments. The results were considered significantly different when ∗*P* < 0.05, ∗∗*P* < 0.01, and ∗∗∗*P* < 0.001.

## Results and Discussion

### Antitumor Activity of *T. spiralis* Infective LE

Conventional treatments of cancers (surgery to remove the tumor mass, chemical drugs, and radiotherapy) have limited efficacies with adverse side effects, such as off-target toxicity and damage to normal tissues and cells (Chidambaram et al., 2011; Nurgali et al., 2018). Consequently, allied agents that help to improve conventional treatment efficiency are sought. Previous evidence has demonstrated that growth inhibition of several types of cancer cells was attributable to *T. spiralis* infection (Lubiniecki and Cypess, 1975; Molinari et al., 1979; Kristek et al., 2005; Wang et al., 2009; Gong et al., 2011; Kang et al., 2013). Therefore, in the present study, the antitumor effects of the *T. spiralis* infective LE on other cancer cells (HepG2 hepatocellular carcinoma, SK-OV-3 ovarian cancer, and A549 lung adenocarcinoma) were investigated for verification and extension of the previous notations on *T. spiralis* anticancer activity.

**Figure 1:**
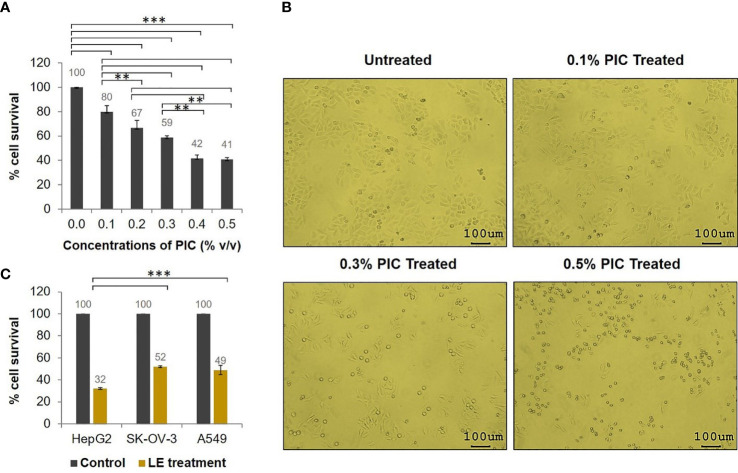
Antitumor activity of the *T. spiralis* infective larval extracts (LE). HepG2, SK-OV-3, and A549 cells (5 × 10^3^ cells) in individual wells of a 96-well culture plate were treated with 70 µgmL^-1^ of *T. spiralis* LE for 24 h. **(A)** Percentage cell survival of HepG2 cells at 24 h posttreatment with different concentrations of PIC. **(B)** Morphological alteration and proliferation of HepG2 cells at 24 h after treatment with different concentrations of PIC visualized at 100× microscopic magnification. **(C)** Percentage cell survivals of HepG2, SK-OV-3, and A549 cells at 24 h posttreatment with 70 µgmL^-1^ of LE compared with the control (cells treated with medium supplemented with 0.05% PIC). The results are shown as the mean ± standard deviation (SD) of three independent experiments. ^∗ ∗^ *P* < 0.01; ^∗ ∗ ∗^ *P* < 0.001.

Because the *T. spiralis* ML extract (LE) contained PIC, different concentrations of PIC were first evaluated to determine a dose that did not affect tumor cell proliferation. By using HepG2 cells as a tested model, it was found that PIC at a 0.1% concentration had the least effect on tumor cell growth (**Figure 1A**), *i.e*., the percentage cell survival of the HepG2 cells exposed to 0.1% PIC was 80%. Nevertheless, the HepG2 cells exposed to 0.1% PIC showed normal morphology compared with the untreated cells, while those treated with higher concentrations of PIC became rounded, small, and did not attach to the plastic surface of the culture flask (**Figure 1B**). The viable cell numbers of HepG2 cells exposed to higher concentrations of PIC were drastically decreased (**Figure 1A**). Given these results, PIC at less than 0.1% concentration (0.05%) was used for the further experiments.

To test the antitumor activity of *T. spiralis* LE, a tumor cell proliferation assay was performed. The results revealed that survival of HepG2, SK-OV-3, and A549 cells treated with 70 µgmL^-1^ of the LE for 24 h was significantly reduced compared to that of the control (cells treated with medium supplemented with 0.05% PIC; **Figure 1C**). The percentage cell survivals of the HepG2, SK-OV-3, and A549 cells after treatment were 32%, 52%, and 49%, respectively, compared with the control cells. These data demonstrated that LE of *T. spiralis* had antitumor activity against HepG2, SK-OV-3, and A549 cells. It exerted the greatest antitumor effect on HepG2 cells compared with other cancer cells (*P* < 0.001).

### Effect of Infective LE on HepG2 Cell Proliferation

**Figure 2:**
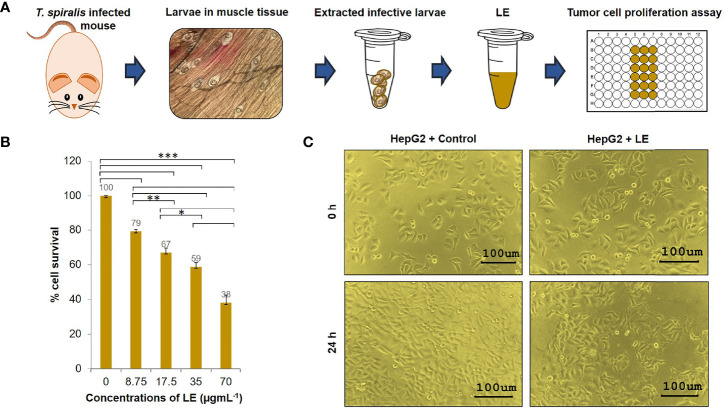
Cytotoxic effects of the infective larval extract (LE) of *T. spiralis* on human hepatocellular carcinoma HepG2 cells. **(A)** Preparation of the LE from *T. spiralis* infective larvae collected from infected mice. **(B)** Percentage cell survivals of HepG2 cells at 24 h posttreatment with different concentrations (8.75, 17.5, 35, and 70 µgmL^-1^) of LE. The results are shown as the mean ± standard deviation (SD) of three independent experiments. ^∗^ *P* < 0.05; ^∗ ∗^ *P* < 0.01; ^∗ ∗ ∗^ *P* < 0.001. **(C)** Proliferations and morphological changes in HepG2 cells treated with LE (70 µgmL^-1^) compared with untreated cells at 24 h. The cells were visualized microscopically at 200× magnification.

Given that LE was most effective against HepG2 cells, the effects of LE on these cells were investigated further. Different concentrations (8.75, 17.5, 35, and 70 µgmL^-1^) of LE were tested for anticancer activity against HepG2 cells using a cell proliferation assay (**Figure 2A**). After a 24-h treatment, growth inhibition of HepG2 cells was found in an LE dose-dependent manner (**Figure 2B**). At 35 µgmL^-1^, LE inhibited the growth of HepG2 cells by approximately 50%. More than 60% of HepG2 cells treated with 70 µgmL^-1^ of LE were dead. The anticancer activity of LE was more prominent at higher than lower dosages. Using probit analysis, the IC_50_ of *T. spiralis* LE on HepG2 growth inhibition was determined to be 44 µgmL^-1^.

In addition, microscopic observation revealed that by 24 h of LE treatment, the HepG2 cells had undergone morphological changes. Specifically, the cells had lost volume (shrinkage), their growth had been retarded, and subcellular organelles had ruptured (**Supplementary Figure 1**). These morphological changes were the same as the changes in HepG2 cells that were exposed to sorafenib (a liver cancer drug) and a methanol extract of the leaf of Indian Head Ginger (*Costus speciosus*) reported previously (Nair et al., 2014). In contrast, HepG2 cells treated with medium supplemented with 0.05% PIC were healthy and had normal growth (**Figure 2C**). These results indicated that LE had antiproliferative and killing activities on human hepatocellular carcinoma HepG2 cells.

### Proteomics of *T. spiralis* Infective LE

**Figure 3:**
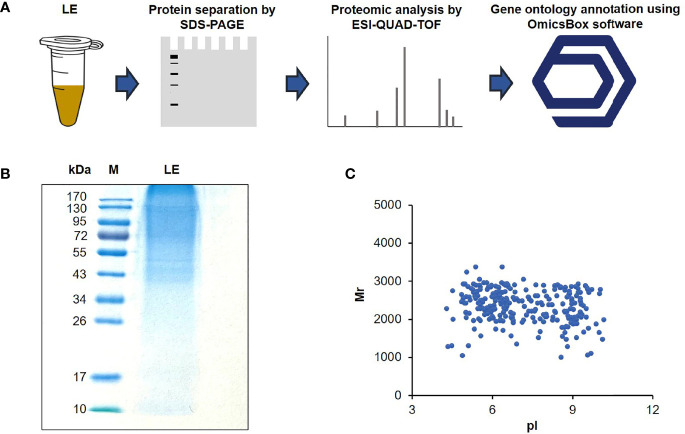
Protein content of the *T. spiralis* infective larval extract (LE). **(A)** Flow diagram of proteomic analysis of LE using electrospray ionization quadrupole ion mobility time-of-flight mass spectrometry (ESI-QUAD-TOF) and OmicsBox software. **(B)** Protein patterns of the LEs on a 12% gel after SDS-PAGE and CBB staining. M, protein marker; numbers on the left are protein molecular masses in kDa. **(C)** Two-dimensional protein relative molecular masses (Mr) and the pH value at which the total charge on each protein was zero (pI).

Since the LE of *T. spiralis* exerted detrimental effects on HepG2 cells, this finding persuaded us to investigate the anticancer components of LE further to find novel antitumor proteins/peptides. For this experiment, *T. spiralis* LE (10 µg) was subjected to sodium dodecyl sulfate-polyacrylamide gel electrophoresis (SDS-PAGE) and stained with CBB (**Figure 3A**). It was found that the *T. spiralis* LE showed several protein bands with molecular weights ranging from 34 to > 170 kDa (**Figure 3B**). The result conformed with those reported previously, in which predominant protein bands of *T. spiralis* ML ranged from 35 to > 116 kDa (Wang et al., 2018). The gel containing the LE proteins was digested by trypsin and submitted to ESI-QUAD-TOF. A total of 270 proteins were identified; 146 proteins (54.1%) had pI 4–7 (**Figure 3C**).

Using BLAST of OmicsBox software, most of the identified proteins hit to *T. spiralis* proteins from the NCBI database (**Table 1**), while the others matched other species of the genus *Trichinella* (**Supplementary Figure 2A**). To understand the functions of the identified proteins, gene ontology (GO) annotation was performed using the Blast2GO tool of OmicsBox software. The 270 identified proteins were grouped into three hierarchically structured GO terms: cellular components, biological processes, and molecular functions. Based on the cellular component classification, 32%, 25%, and 16% of the identified proteins were localized to the intracellular anatomical structure, organelle, and membrane, respectively (**Supplementary Figure 2B**). Classification based on the biological process functions revealed that these proteins were relevant to organic substance metabolic processes (17%), primary metabolic processes (17%), nitrogen compound metabolic processes (16%), and cellular metabolic processes (16%) (**Supplementary Figure 2C**). Moreover, these proteins were found to have diverse molecular functions, such as ion binding (15%), protein binding (15%), organic cyclic compound binding (15%), and heterocyclic compound binding (15%) (**Supplementary Figure 2D**). These data indicate that various proteins in the extract of ML play critical roles in several key *T. spiralis* life cycle processes.

**Table 1:** Forty-four different proteins of muscle larval extract (LE) that hit to *Trichinella spiralis* proteins.

No.	Protein name	Accession no.	Theoretical Mr	Theoretical pI
1	3-hydroxypropionyl-coenzyme A dehydratase	KRY34169.1	1661.8957	9.42
2	43 kD-secreted glycoprotein	AAA30327.1	2193.1722	5.95
3	Acetylcholinesterase	KRY36039.1	2925.5577	6.43
4	Ankyrin-2	KRY39858.1	2750.2745	4.51
5	Cysteine-rich motor neuron 1 protein	KRY34257.1	2871.2561	6.12
6	Derlin-2	KRY38484.1	2600.2462	5.34
7	DNA mismatch repair protein Msh2	KRY36726.1	2577.2394	6.23
8	E3 ubiquitin-protein ligase UBR5	KRY34630.1	2272.1481	6.51
9	Enoyl-coA hydratase/isomerase family protein	XP_003381315.1	2808.2340	9.20
10	Eukaryotic translation initiation factor 2-alpha kinase 4	KRY32451.1	2894.3864	5.96
11	Forkhead box protein A2-A	KRY36091.1	2695.4870	6.19
12	Heat shock protein beta-1	KRY32883.1	1676.8370	7.79
13	Histone acetyltransferase KAT2A	KRY37009.1	2341.0588	7.20
14	Huntingtin	KRY40707.1	2960.4493	6.34
15	Hypothetical protein T01_12692	KRY24797.1	2283.0412	5.55
16	Hypothetical protein T01_13886	KRY27735.1	2059.0685	8.26
17	Hypothetical protein T01_3439	KRY38294.1	2214.9606	8.32
18	Hypothetical protein T01_4238	KRY29200.1	2063.0358	8.03
19	Hypothetical protein T01_4307	KRY40518.1	2619.2586	7.60
20	Hypothetical protein T01_7381	KRY43100.1	1891.0210	9.08
21	Hypothetical protein T01_7775	KRY36588.1	2103.9709	6.28
22	Hypothetical protein T01_9093	KRY30334.1	2095.9390	8.82
23	Insulin receptor	KRY41238.1	2587.1578	7.34
24	Leucine-rich repeat-containing protein let-4	KRY32860.1	1973.9364	7.16
25	Ligand-gated ion channel family protein	XP_003376620.1	2486.0253	6.44
26	LIM domain-containing protein unc-97	KRY35281.1	2886.3081	9.48
27	Nuclear pore complex protein	KRY39624.1	2745.2659	5.44
28	Phosphatidylinositol phosphatase PTPRQ	KRY42984.1	2214.1157	7.35
29	Poly-cysteine and histidine-tailed protein	KRY34608.1	2383.1055	8.22
30	Prion-like-(Q/N-rich) domain-bearing protein 25	KRY38806.1	2584.0741	7.35
31	Prolow-density lipoprotein receptor-related protein 1	KRY39792.1	2771.3048	5.28
32	Protein disulfide-isomerase 2	KRY37705.1	1309.7030	5.10
33	Protein VPRBP	KRY32611.1	2444.3865	5.62
34	Putative immunoglobulin I-set domain protein	XP_003374402.1	2934.4363	4.92
35	Putative integrase core domain protein	XP_003369623.1	2043.1261	8.95
36	Putative low-density lipoprotein receptor domain class A	XP_003371813.1	2701.2193	5.38
37	Serine/threonine-protein kinase mig-15	KRY38922.1	2909.4885	6.83
38	Serine/threonine-protein kinase PCTAIRE-2	XP_003372037.1	2711.2472	9.60
39	Serine/threonine-protein kinase SMG1	KRY38287.1	2538.1242	5.72
40	Small G protein signaling modulator 3-like protein	XP_003376050.1	2418.9991	9.32
41	Striatin-interacting protein 2	KRY28237.1	2142.1039	6.78
42	T-complex protein 1 subunit theta	KRY36024.1	2475.3009	6.30
43	Titin	KRY34562.1	2430.1658	5.00
44	Tubulin-specific chaperone D	KRY36124.1	2507.1494	6.54

**Figure 4:**
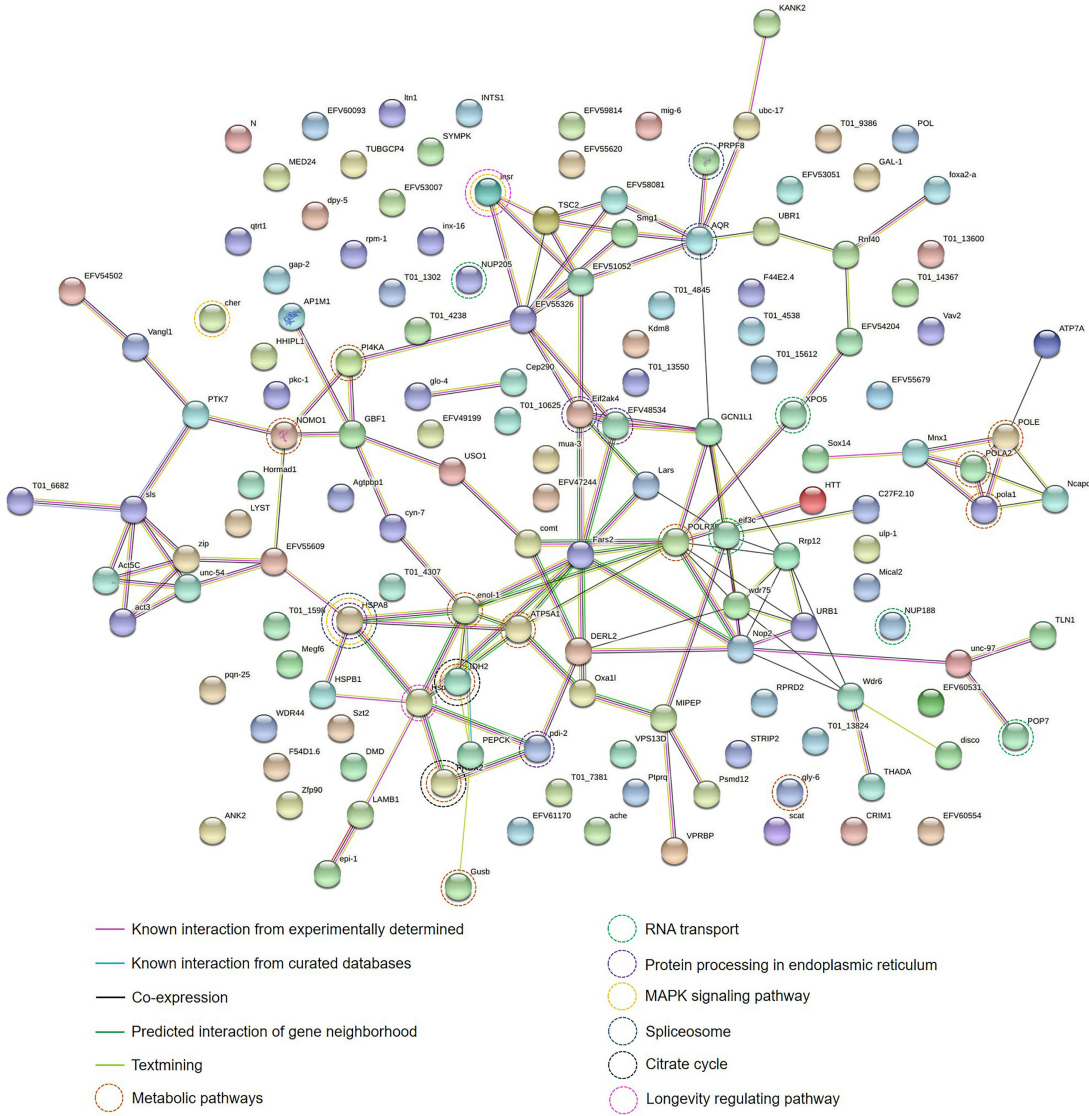
Protein-protein interaction network of identified proteins of the muscle larval extract (LE) of *Trichinella spiralis* using the STRING database. The types of interactions are represented by different colored lines (lower left panel). The functions of each protein-encoding gene are represented by different colored circle lines (lower right panel).

To understand the protein-protein interactions among the 270 identified proteins along with their putative pathways, STRING analysis was performed. Only 146 of 270 identified proteins could be analyzed based on the database of *T. spiralis*. The protein-protein interaction network showed that most identified proteins were interconnected by the experimental database (**Figure 4**). POLE, NOMO1, IDH2, PEPCK, POLR3B, ATP5A1, gly-6, Gusb, POLA2, enol-1, pola1, and PI4KA gene products play roles in metabolic pathways, including pyrimidine metabolism, purine metabolism, and DNA replication, of *T. spiralis*. NUP205, NUP188, XPO5, POP7, and eif3c gene products are pivotal in RNA transport. Moreover, HSPA8, cher, and insr gene products play a crucial role in the mitogen−activated protein kinase (MAPK) signaling pathway. The MAPK cascades are key signaling pathways that regulate a broad range of cellular processes, including proliferation, differentiation, apoptosis, and stress responses (Guo et al., 2020). In particular, the MAPK cascades play a central role in angiogenesis and tumor metastasis (Guo et al., 2020).

### Antitumor Peptide Candidates Analyzed by *In Silico* Analysis

The use of therapeutic peptides as cancer drugs has recently attracted attention (Tyagi et al., 2013). Numerous advantages of this kind of therapy have been ascribed to high tumor penetration, high specificity, ease of synthesis and modification, and low production cost of the peptides (Vlieghe et al., 2010). Tyagi et al. (2013) found that Cys, Gly, Ile, Lys, and Trp are dominant at various positions in general anticancer peptides. Therefore, these amino acids were used as the key features to predict anticancer peptides in conjunction with other databases and experimental validation of the *in silico* data. A model for prediction called AntiCP, a support vector machine (SVM)-based mode, was developed (Agrawal et al., 2021).

In the present investigation, 44 identified LE proteins that matched to the *T. spiralis* protein orthologs of the database were predicted for the anticancer peptides using AntiCP and ACPred-FL webservers. Among the 44 proteins, the antitumor function of three anticancer peptides was interesting. These were peptides matching to partial sequences of 3-hydroxypropionyl-coenzyme A dehydratase (named antitumor peptide 1), hypothetical protein T01_4238 (named antitumor peptide 2), and putative low-density lipoprotein receptor domain class A (named antitumor peptide 3) of *T. spiralis* (**Tables 1** and **2**). Antitumor peptides 1 and 2 have SVM and prediction scores ≥ 0.80 and > 95% confidence by the AntiCP and ACPred-FL algorithms, respectively (**Table 2**).

**Table 2:** Prediction of antitumor peptides of 44 proteins identified from muscle larval extract (LE) using AntiCP and ACPred-FL prediction tools.

No.	Peptide sequence	AntiCP			ACPred-FL
		SVM score	No. of + motifs	No. of - motifs	Prediction result (% confidence)
1	MIGLSRALDMIITGR	0.80	0	0	Yes (96.89)
2	TATDDTEWFLLFKPVGLLK	0.72	0	1	Yes (99.22)
3	TAEMISRIVVSIAVCHHLLLVICCFR	0.77	0	2	Yes (92.55)
4	STEDYEHHPDPETALPAMEVLELK	0.68	0	1	Yes (97.98)
5	MDTGVCHLSGVSYNIGEMWRDNICR	0.68	0	1	Yes (92.55)
6	VLAMLVSSEELSEYCRVLDDTR	0.76	0	0	Yes (92.55)
7	FGGEKSADNLYGILNHCQTAVGQR	0.71	0	2	No (94.17)
8	EPIESISCSSVRTTVLTTHR	0.77	0	1	No (79.97)
9	MMVCVGNNFNNRSSVFVVCIDDEK	0.70	0	1	Yes (92.55)
10	AFMENCSVTESSMQQKFLLTLSSLK	0.67	0	2	No (60.63)
11	SELAVCAPPDNIAEELIKAILFLVK	0.76	0	1	Yes (92.55)
12	GVKPEQFTSNLSADGK	0.34	0	0	Yes (98.11)
13	MLYHCIFMSLRHCISEGR	0.75	0	0	Yes (92.55)
14	AWSTTTTVNGKCLACPLMQHCLQLLR	0.66	0	1	No (86.13)
15	FLATDSNCTQLEWMRGNPK	0.71	0	1	No (60.63)
16	TPSDKGIPISTTDATLSVEK	0.66	0	0	Yes (96.89)
17	AQWQAYEEAWNSNDYAAAK	0.82	0	1	No (79.97)
18	SGILSSFQIVLDDASCPVR	0.83	0	0	Yes (99.22)
19	NVDTSDGLSLTIDALPTTCPVSSEK	0.72	0	2	Yes (99.22)
20	NLNLHTMTLRSAVLHR	0.70	0	0	Yes (99.22)
21	LTVNGNYRPANDDDLLEAD	0.76	0	0	Yes (99.22)
22	ARLCFACLEPGHYASGCK	0.78	0	1	No (94.17)
23	SENCSHVTAMTTTTSCLFGRNVR	0.76	0	0	Yes (97.98)
24	QRYLSMLIGSDSTSDSAK	0.80	0	1	Yes (99.22)
25	QSYCYCIIYDENEEDASLSK	0.78	0	1	Yes (96.89)
26	CFVCAQCFRPFPDGIFFEFESRK	0.73	0	0	No (86.13)
27	MVSFELEQMLTVEGINDMQRDTK	0.77	0	1	Yes (97.98)
28	NRTPTSLHVMWIPFWGTR	0.74	0	1	Yes (97.98)
29	KFMFHYNFASFATNELSSAR	0.60	0	0	Yes (94.40)
30	AKSRPGGSCEHGEECTDGSYCLK	0.80	0	0	No (94.17)
31	DGLVDTFVGNCAGMSIDWVAGNFVLR	0.72	0	0	No (94.17)
32	VLTGNNFASFIK	0.76	0	0	Yes (94.40)
33	LYLIFKNLPDDNQMILIILR	0.70	0	0	Yes (96.89)
34	LLDLPDNCLLHILENLTIMQCTYCK	0.76	0	2	Yes (97.98)
35	FPALLPCKGMIVDLLIER	0.80	0	0	No (94.17)
36	NPCGQNNGGCSHLCLLVSDQKAVCK	0.70	2	1	Yes (87.23)
37	TIDDQFLWGSSLMIVPILEPYSTLR	0.66	0	1	Yes (97.98)
38	LNQGGLLFFGENEADHCFCSLNVK	0.65	0	0	Yes (96.89)
39	MMYRCYTNIDPQAHGLGSFYR	0.73	0	0	Yes (96.89)
40	EKFPDADPEHCNGTCCRPESK	0.67	0	0	No (79.97)
41	LAALKCLAYLLQGAFMECK	0.76	0	0	Yes (92.55)
42	IGVAIDLIQQILFDDGNQVNYK	0.77	0	0	Yes (99.22)
43	CVITNIIGSDETSCKVTVEEYK	0.73	0	1	No (86.13)
44	AVVDLCCEFMQYTKLQAASESR	0.73	0	1	No (79.97)

+, positive; -, negative.

The peptide that matched to putative low-density lipoprotein receptor domain class A was the third antitumor candidate. Even though this peptide’s SVM and prediction scores were lower than 0.80 and 95% confidence when analyzed by the AntiCP and ACPred-FL algorithms, respectively, two positive motifs of this peptide were found by AntiCP prediction (**Table 2**). However, a peptide that matched to leucine-rich repeat-containing protein let-4 was not chosen for antitumor activity testing even though the prediction by AntiCP and ACPred-FL algorithms gave SVM and the prediction scores at ≥ 0.80 and > 95% confidence. This was because one negative anticancer peptide motif was found through AntiCP prediction (**Table 2**).

Among the three antitumor peptide candidates chosen, 3-hydroxypropionyl-CoA dehydratase is a member of the enoyl-CoA hydratase family reported to be associated with the progression and metastasis of cancers (Zhang et al., 2015). This family can function as either a tumor promoter or a tumor suppressor for certain cancers under different circumstances (Zhang et al., 2015). The function of the hypothetical protein T01_4238 of *T. spiralis* is still unknown. However, using BLASTP analysis, it was found that the amino acid sequence of this protein shared identity with the multicystatin-like domain protein precursor of *T. spiralis* (28.39% identity and 90% query coverage). This protein is secreted from *T. spiralis* ML and was defined as a critical immunomodulatory factor contributing to the immune evasion strategies of the parasite (Robinson et al., 2007). This evidence highlights that the peptide might have potential as a novel antitumor drug for hepatocellular carcinoma treatment. An experimental investigation is needed to validate this speculation.

### Anti-Hepatocellular Carcinoma Effects and Molecular Mechanisms of the Antitumor Peptides

**Figure 5:**
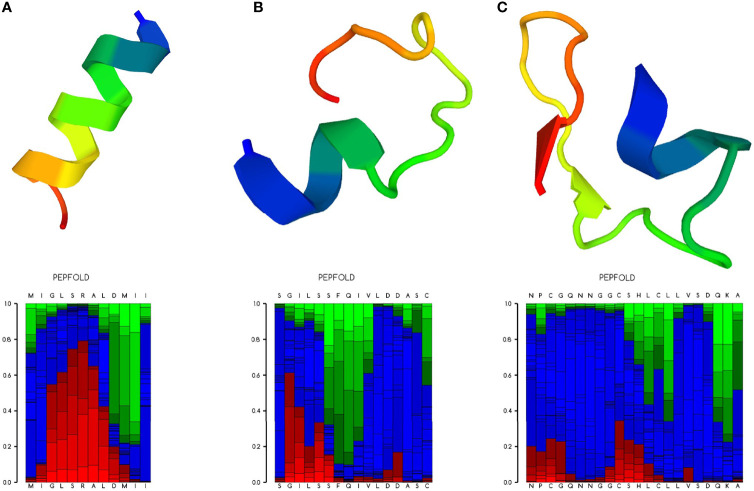
Ribbon diagram of the selected antitumor peptide candidates. Amino acid sequences of the three peptide candidates were predicted for their peptide structures using PEP-FOLD3 (Lamiable et al., 2016). **(A)** Predicted peptide structure (upper panel) with the local structure prediction profile (lower panel) of antitumor peptide 1. **(B)** Predicted peptide structure with the local structure prediction profile of antitumor peptide 2. **(C)** Predicted peptide structure with the local structure prediction profile of antitumor peptide 3. The profile is presented using the following color codes: red, helical; green, extended; and blue, coil.

**Figure 6:**
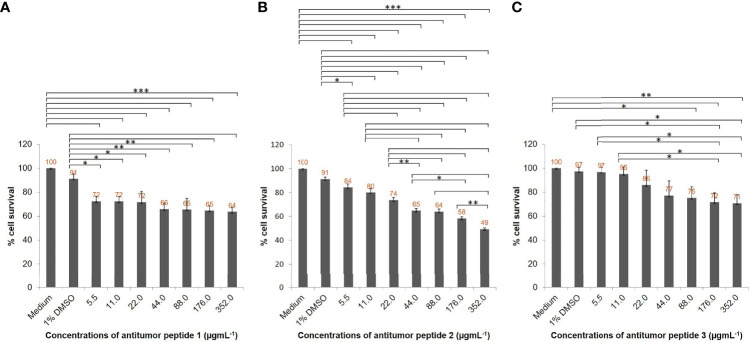
Antihepatocellular carcinoma activity of the three peptide candidates. HepG2 cell lines (5 × 10^3^ cells) in individual wells of a 96-well culture plate were treated with different concentrations (5.5, 11, 22, 44, 88, 176, and 352 µgmL^-1^) of individual antitumor peptides for 24 h. **(A)** Percentage cell survivals of HepG2 cells at 24 h posttreatment with different concentrations of antitumor peptide 1. **(B)** Percentage cell survivals of HepG2 cells at 24 h posttreatment with different concentrations of antitumor peptide 2. **(C)** Percentage cell survivals of HepG2 cells at 24 h posttreatment with different concentrations of antitumor peptide 3. Medium alone and medium supplemented with 1% DMSO were used as negative and diluent controls, respectively. The results are shown as the mean ± standard deviation (SD) of three independent experiments. ^∗^ *P* < 0.05; ^∗ ∗^ *P* < 0.01; ^∗ ∗ ∗^ *P* < 0.001.

To verify the antihepatocellular carcinoma activities of the three peptide candidates, peptides with 15 amino acids (antitumor peptide 1; MW: 1.87 kDa; MIGLSRALDMIITGR; α-helical peptide; **Figure 5A**), 19 amino acids (antitumor peptide 2; MW: 2.23 kDa; SGILSSFQIVLDDASCPVR; β-pleated sheet peptide; **Figure 5B**), and 25 amino acids (antitumor peptide 3; MW: 2.81 kDa; NPCGQNNGGCSHLCLLVSDQKAVCK; β-pleated sheet peptide; **Figure 5C**) were synthesized, and a tumor cell proliferation assay was performed as described above. The antitumor activities of the three peptides are illustrated in **Figures 6A** **–C**. The results revealed that antitumor peptide 2 had the greatest antitumor effect and displayed dose-dependent activity against HepG2 cells (**Figure 6B**).

HepG2 cells treated with different concentrations of antitumor peptide 2 had significantly reduced viability at 24 h compared with the negative control (cells treated with medium alone) and diluent control (cells in medium containing 1% DMSO; **Figure 6B**). The percentage cell survivals of HepG2 cells after treatment with 88, 176, and 352 µgmL^-1^ antitumor peptide 2 were 64%, 58%, and 49%, respectively. The affected cells shrank, did not proliferate, and exhibited subcellular organelle rupture (**Supplementary Figure 4**). Using probit analysis, this peptide’s HepG2 inhibitory concentration 50 (IC_50_) was determined to be 337 µgmL^-1^. Antitumor peptides 1 and 3 had less inhibitory activity against HepG2 cells than antitumor peptide 2 (**Figures 6A** **, C** and **Supplementary Figures 3**, **5**). At 352 µgmL^-1^ of antitumor peptides 1 and 3, the percentage cell survivals of HepG2 cells were 64% and 71%, respectively. The IC_50_ values of antitumor peptides 1 and 3 toward HepG2 cells were 73 152 and 916 µgmL^-1^, respectively.

To explore the molecular function of antitumor peptide 2, peptide-mediated HepG2 cell apoptosis and necrosis were investigated using Annexin V/propidium iodide (PI) staining and flow cytometric analysis. Usually, phosphotidylserine translocates to the outer leaflet of a cell membrane in the early stage of the apoptotic process (Nagata et al., 2016). Annexin V is a green fluorescence that selectively binds to phosphotidylserine (Demchenko, 2013). Hence, an increase in Annexin V-stained cells indicates that the cells are undergoing apoptosis. Propidium iodide (PI) is a red fluorescence that intercalates in double-stranded DNA when the cells undergo necrosis (Nescerecka et al., 2016). An increase in the number of PI-stained cells indicates cellular necrosis.

**Figure 7:**
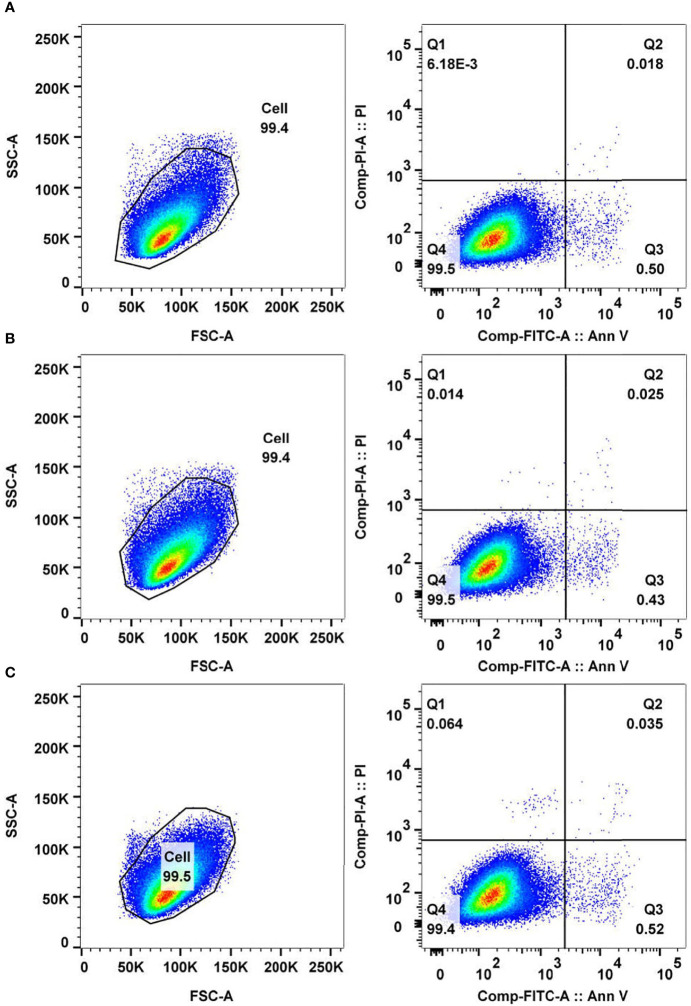
Analysis of cancer cell apoptosis and necrosis mediated by antitumor peptide 2. HepG2 cells (5 × 10^5^ cells) were treated with 337 µgmL^-1^ of antitumor peptide 2, 1% DMSO (diluent control), and medium alone (negative control) for 24 h. Cells of all treatments were stained with fluorochrome-conjugated Annexin V and propidium iodide (PI) and analyzed by flow cytometry. **(A)** FACS analyses of Annexin V- and PI-stained HepG2 cells treated with antitumor peptide 2. **(B)** FACS analyses of Annexin V- and PI-stained HepG2 cells treated with 1% DMSO. **(C)** FACS analyses of Annexin V- and PI-stained HepG2 cells treated with medium alone. Lower right quadrant (Q3) Annexin V-positive/PI-negative cells denote early apoptotic cells, and upper right quadrant (Q2) Annexin V-positive/PI-positive cells denote necrotic or late apoptotic cells.

In the current study, it was found that the number of Annexin V-stained cells (lower right quadrant, Q3) and Annexin V/PI-stained cells (upper right quadrant, Q2) among the antitumor peptide 2-treated HepG2 cells was not significantly different from that of the controls (**Figure 7**). The data suggested that antitumor peptide 2 did not induce apoptosis or necrosis in HepG2 cells and may have inhibited HepG2 cell proliferation *via* other mechanisms or pathways apart from types I (apoptosis) and III (necrosis) programmed cell death. Interestingly, it has been reported that many anticancer drugs, including *N*-desmethyldauricine, did not cause apoptosis in cancer cells but induced type II programmed cell death (autophagy) (Law et al., 2017). This probability should be investigated further for antitumor peptide 2.

It was reported that the central mechanism of many therapeutic drugs for cancers, including monoclonal antibodies and tyrosine kinase inhibitors, is the activation of programmed cell death *via* the elevation of reactive oxygen species (ROS) levels within cancer cells (Dimitrov and Marks, 2009; Hartmann et al., 2009; Teppo et al., 2017). Chemotherapy and radiotherapy also increase intracellular ROS, leading to programmed cell death (Shen et al., 2013; Brenneisen and Reichert, 2018). Elevation of ROS levels has been recognized as a basis for developing new therapeutic agents and strategies for cancers (Perillo et al., 2020).

**Figure 8:**
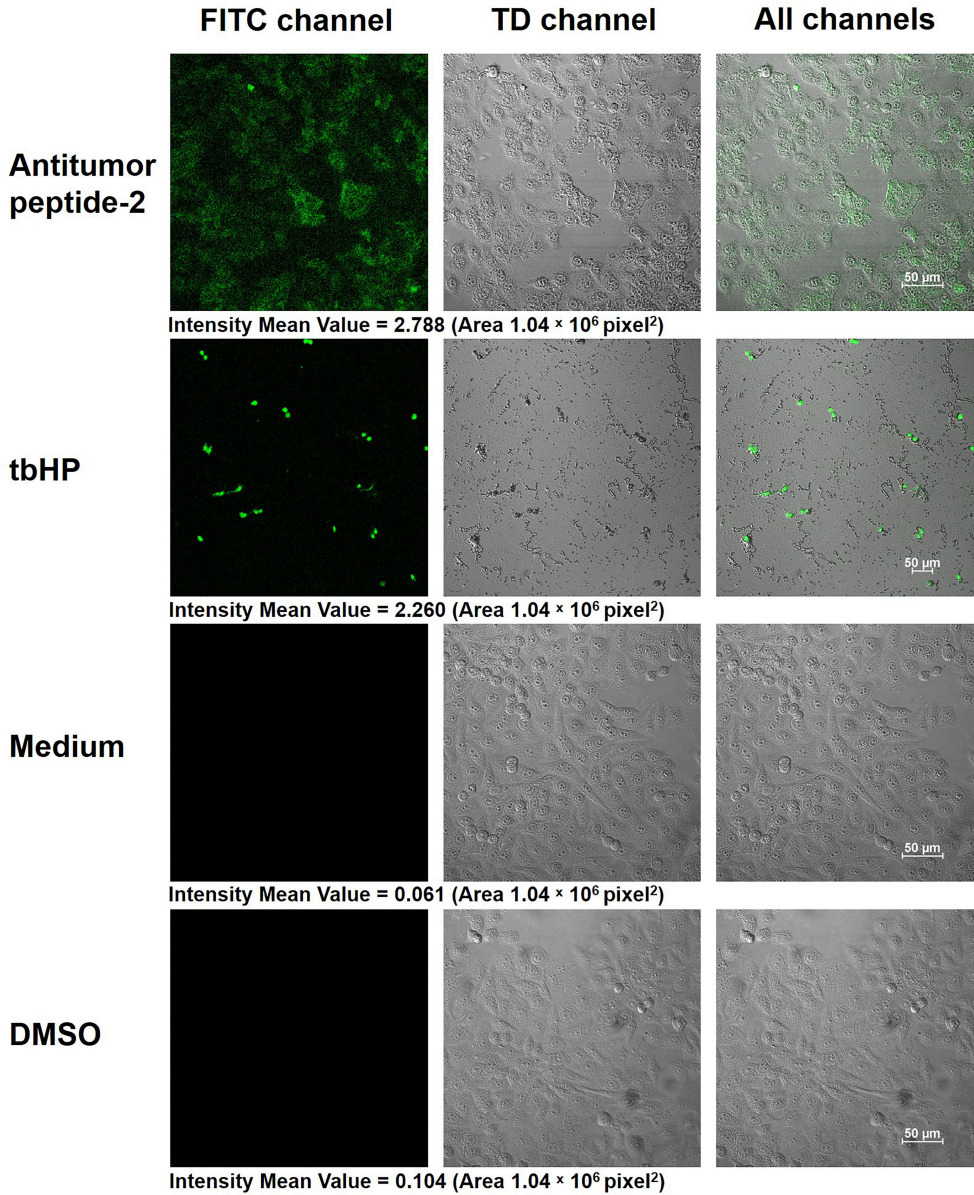
Production of reactive oxygen species (ROS) within HepG2 cells after antitumor peptide 2 treatment. HepG2 cells (1 × 10^5^ cells) were treated with 337 µgmL^-1^ of antitumor peptide 2, 50 µM of tert-butyl hydrogen peroxide (tbHP; positive control), medium alone (negative control), and 1% DMSO (diluent control) for 24 h. All cells were stained with 2’, 7’-dichlorofluorescin diacetate (DCFDA) solution (DCFDA/H2DCFDA-Cellular ROS Assay Kit) and visualized by confocal microscopy at 400× magnification. Images were recorded under FITC, TD, and all channels. Scale bar = 50 μm.

The current work identified increased ROS within HepG2 cells after antitumor peptide 2 treatment using the cell-permeant reagent DCFDA. DCFDA is a fluorogenic dye for measuring ROS activity. After diffusion of DCFDA into the cell, it is deacetylated by cellular esterase to a nonfluorescent compound that ROS later oxidizes into fluoresced 2’, 7’-dichlorofluorescein (DCF), which is detectable by fluorescence microscopy (Rastogi et al., 2010). The data presented here demonstrated that HepG2 cells treated with antitumor peptide 2 had elevated ROS levels within the cells (intensity mean value is 2.788) compared to the medium control (intensity mean value is 0.061), similar to HepG2 cells treated with tbHP, the positive control (intensity mean value is 2.260) (**Figure 8**). The results indicated that antitumor peptide 2 induced ROS accumulation within hepatocellular carcinoma cells, thereby inhibiting cancer cell proliferation.

These data are consistent with the study of Charruyer et al. (Charruyer et al., 2005). They found that the programmed cell death of the human myeloblastic cell line U937 was the effect of elevated ROS on sphingomyelinase after irradiation, which generated ceramide from sphingomyelin and binds to death receptors on the cell membrane of cancer cells (Charruyer et al., 2005). Elevated ROS levels have been reported to cause apoptosis, autophagy, and necrosis of cancer cells (Perillo et al., 2020). For autophagy induction, ROS induced the formation of LC3-dependent autophagosomes by suppressing TORC1, the negative regulator of autophagy (Alexander et al., 2010). Based on these data, one therapeutic approach destroying tumor cells has been presented recently by ROS-induced autophagy (Scherz-Shouval and Elazar, 2011). Rapamycin is an example of an anticancer drug that inhibits Ras-driven tumor cell proliferation by causing oxidative stress and autophagy (De Raedt et al., 2011). Therefore, antitumor peptide 2 presented in the current study has potential as a novel anticancer drug since it increased the ROS level in human hepatocellular carcinoma HepG2 cells and inhibited cell proliferation.

## Conclusions

Data from this study demonstrated that the extract of *T. spiralis* infective larvae (LE) inhibited the proliferation of human hepatoma, ovarian cancer, and lung adenocarcinoma cells, in addition to the other cancer cells reported previously. Among the three types of cancer cells tested, LE exerted the most inhibitory effect on human hepatocellular carcinoma HepG2 cells. Both LE and LE-derived antitumor peptides (identified by proteomics and bioinformatics) showed dose-dependent detrimental effects against HepG2 cells: cellular shrinkage, proliferation retardation, and subcellular organelle rupture. These effects are similar to those mediated by sorafenib, a chemical inhibitor for the treatment of liver cancer. The antitumor activity of the LE-derived peptide did not involve apoptosis or necrosis but rather the elevation of reactive oxygen species within the cells. Although the precise molecular mechanism and pathway of the cancer growth inhibition and killing mediated by the LE-derived peptide await elucidation, the data reported herein provide compelling evidence that LE-derived peptide has inherent antineoplastic activity and should be tested further towards clinical application as an allied agent for cancer therapy.

## Data Availability Statement

The datasets presented in this study can be found in online repositories. The name of the repository and accession number can be found below: PRIDE, EBI; PXD032081.

## Ethics Statement

The animal study was reviewed and approved by Mahidol University-Institute Animal Care and Use Committee (MU-IACUC) of the Faculty of Medicine Siriraj Hospital, Mahidol University (project code, SI-ACUP 007/2562; approval number, 014/2562).

## Author Contributions

PR designed and performed the experiments, collected and analyzed the data, prepared figures and tables, and wrote the original manuscript. OR, LK, and MC investigated and analyzed the data. PA and KK provided resources. WC provided resources, supervised the project, and edited the final draft of the manuscript. All authors approved the final version of the manuscript.

## Funding

This work was supported by a Research Grant for New Scholars (grant number MRG6280087, 2019) from the Thailand Research Fund and the Office of the Higher Education Commission, Thailand.

## Conflict of Interest

The authors declare that the research was conducted in the absence of any commercial or financial relationships that could be construed as a potential conflict of interest.

## Publisher’s Note

All claims expressed in this article are solely those of the authors and do not necessarily represent those of their affiliated organizations, or those of the publisher, the editors and the reviewers. Any product that may be evaluated in this article, or claim that may be made by its manufacturer, is not guaranteed or endorsed by the publisher.
