# Supplementary material for: Peptide of Trichinella spiralis Infective Larval Extract That Harnesses Growth of Human Hepatoma Cells
Source: Front Cell Infect Microbiol. 2022 Apr 26;12:882608. doi: 10.3389/fcimb.2022.882608 (PMC9086976; doi:10.3389/fcimb.2022.882608)
Supplement: Supplementary file 1 [file DataSheet_1.docx]

Supplementary Material

# Supplementary Figures


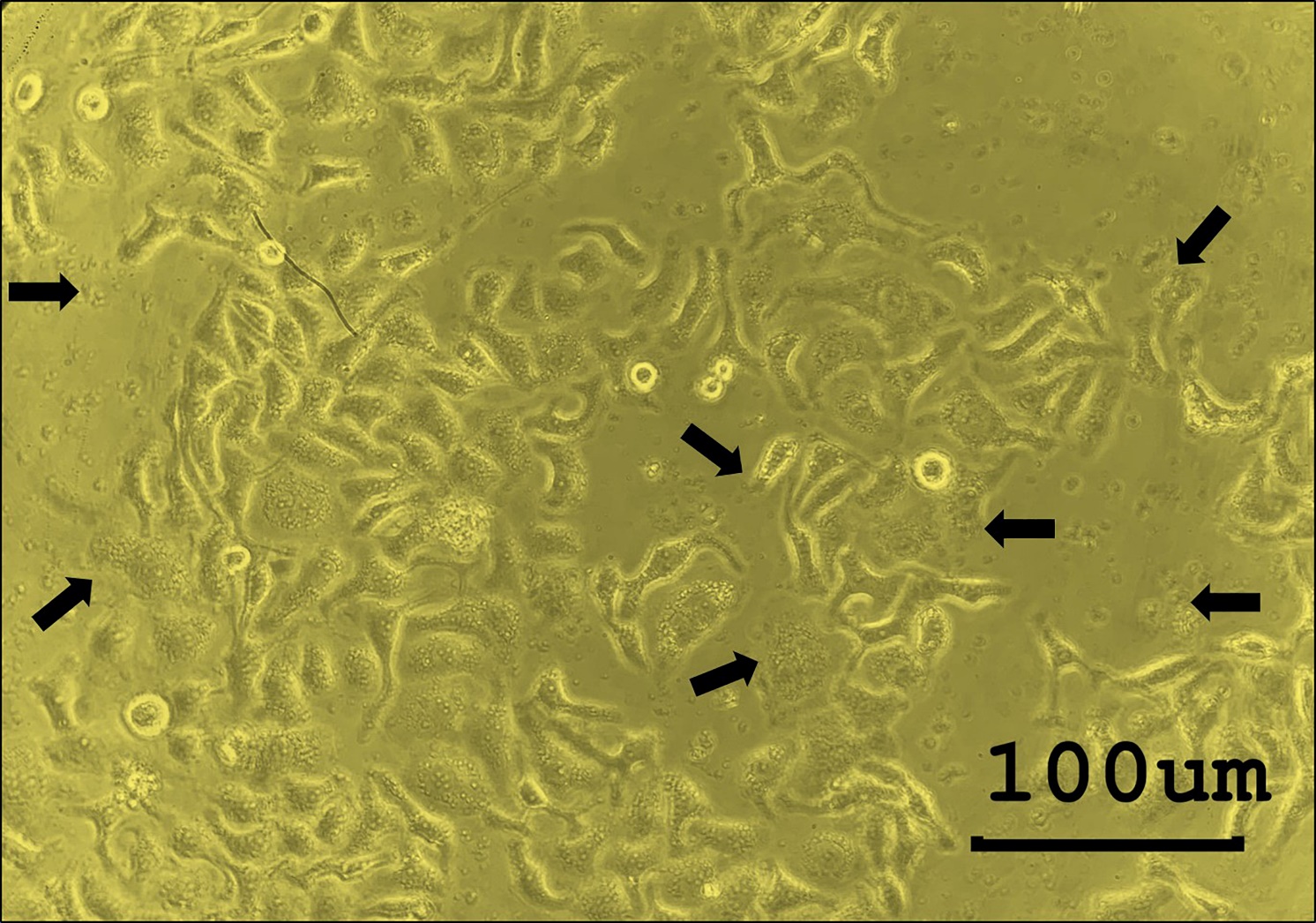


**Supplementary Figure 1.** Morphological change of HepG2 cells that were exposed to larval extract (LE) of *T. spiralis*. Microscopic observation revealed that by 24 h of LE treatment, the HepG2 cells had lost volume (shrinkage), their growth had been retarded, and subcellular organelles had ruptured (indicated by black arrows).

**
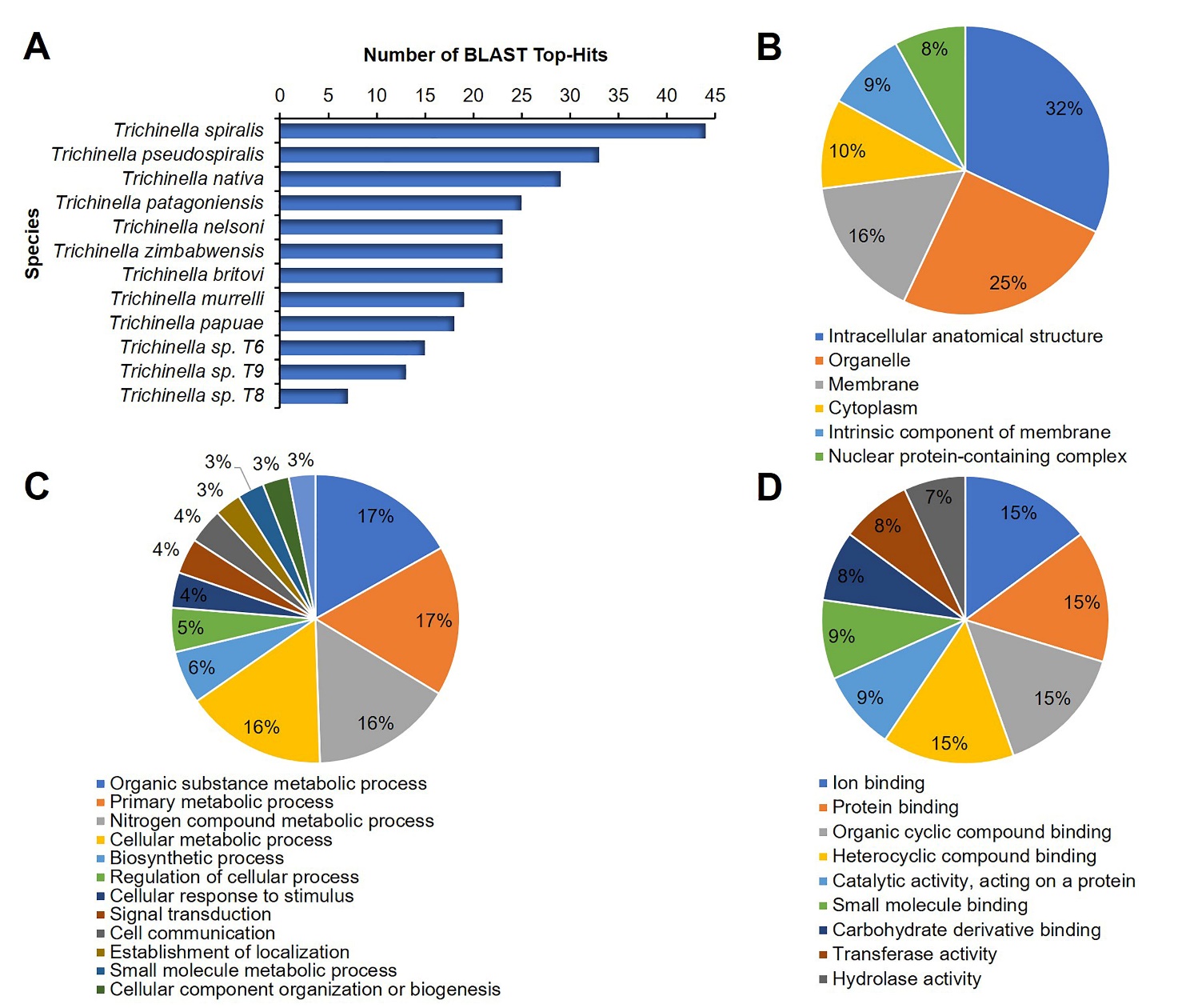
**

**Supplementary Figure 2.** Gene ontology (GO) enrichment analysis of the proteins in infective larval extract (LE) of *T. spiralis*. All identified proteins were blasted to proteins from the NCBI database using BLAST of OmicsBox software (Götz et al., 2008) and categorized into three groups (cellular components, biological process, and molecular function) using GO annotation *via* the Blast2GO tool. **(A)** Number of BLAST top hits of the LE proteins. **(B)** Classification of the LE proteins based on cellular components. **(C)** Classification of the LE proteins based on biological process. **(D)** Classification of the LE proteins based on molecular function.


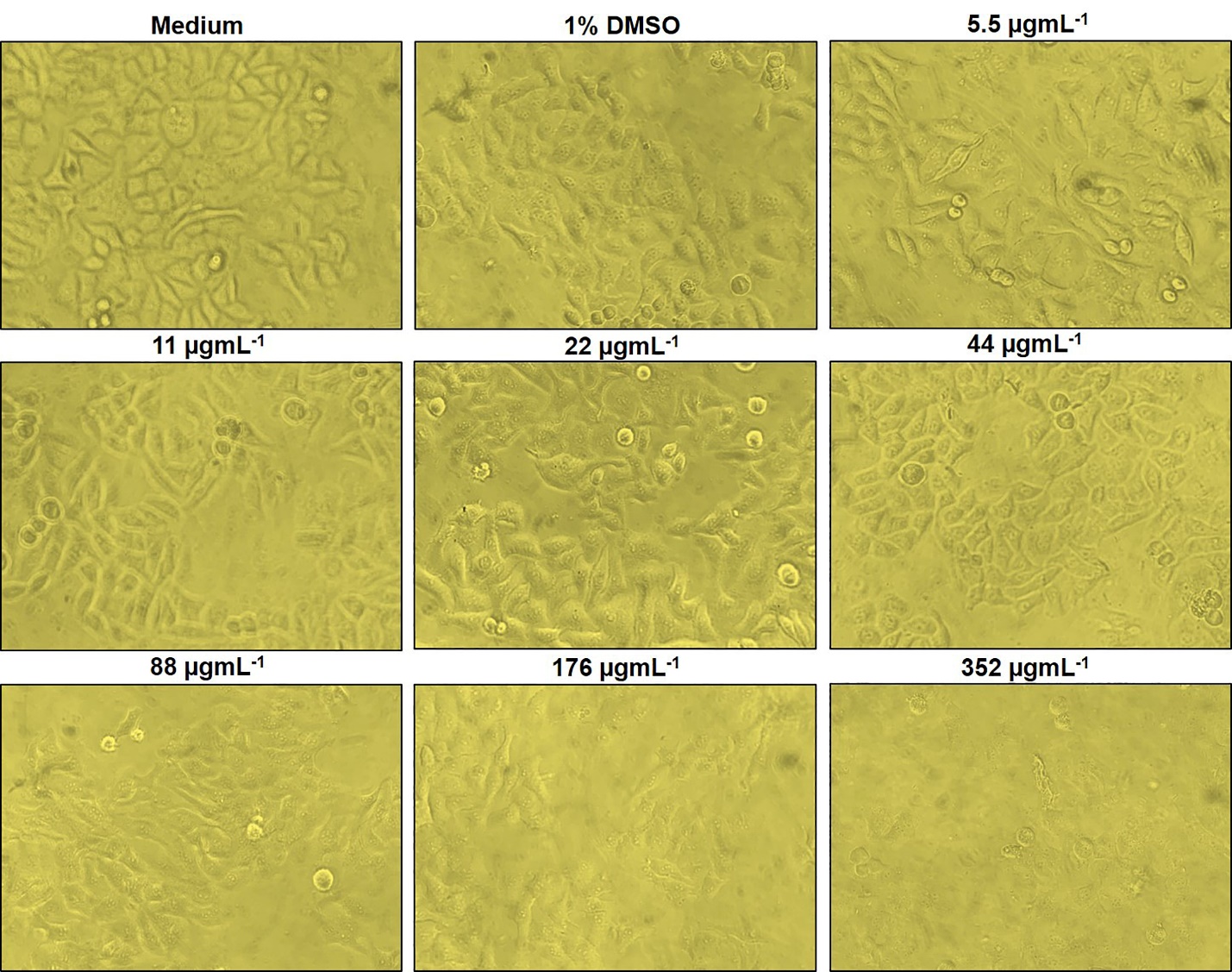


**Supplementary Figure 3.** Cytotoxic effects of antitumor peptide 1 on hepatocellular carcinoma HepG2 cells. Proliferations and morphological changes of HepG2 cells treated with different concentrations (5.5, 11, 22, 44, 88, 176, and 352 µgmL^-1^) of antitumor peptide 1, medium alone (negative control), and medium supplemented with 1% dimethyl sulfoxide (DMSO; diluent control) were observed at 24 h. They were visualized using an Olympus IX70 Inverted Tissue Culture Microscope (Olympus, Tokyo, Japan) at 200× magnification.


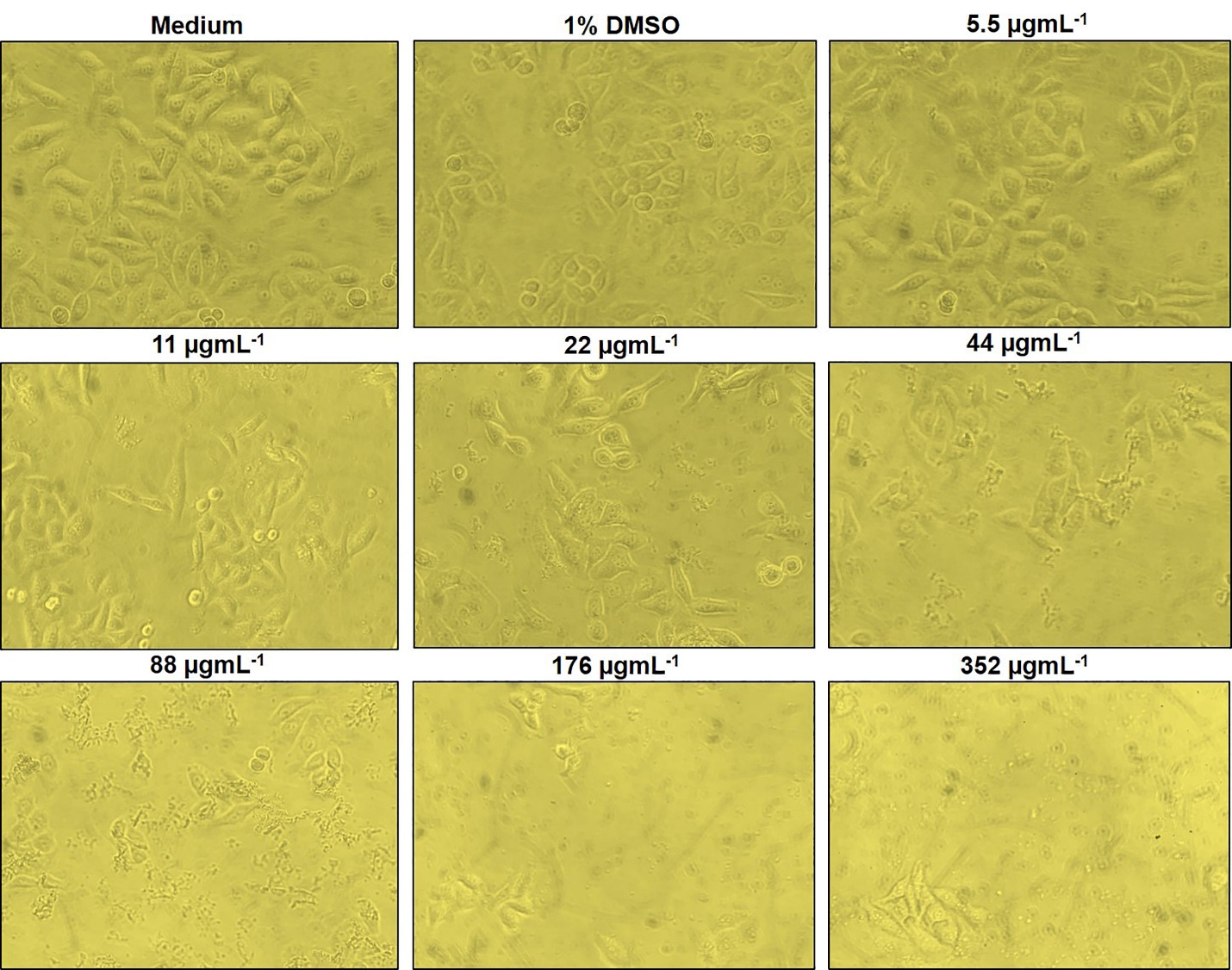


**Supplementary Figure 4.** Cytotoxic effect of antitumor peptide 2 on hepatocellular carcinoma HepG2 cells. Proliferations and morphological changes of HepG2 cells treated with different concentrations (5.5, 11, 22, 44, 88, 176, and 352 µgmL^-1^) of antitumor peptide 2, medium alone (negative control), and medium supplemented with 1% dimethyl sulfoxide (DMSO; diluent control) were observed at 24 h. They were visualized using an Olympus IX70 Inverted Tissue Culture Microscope (Olympus, Tokyo, Japan) at 200× magnification.


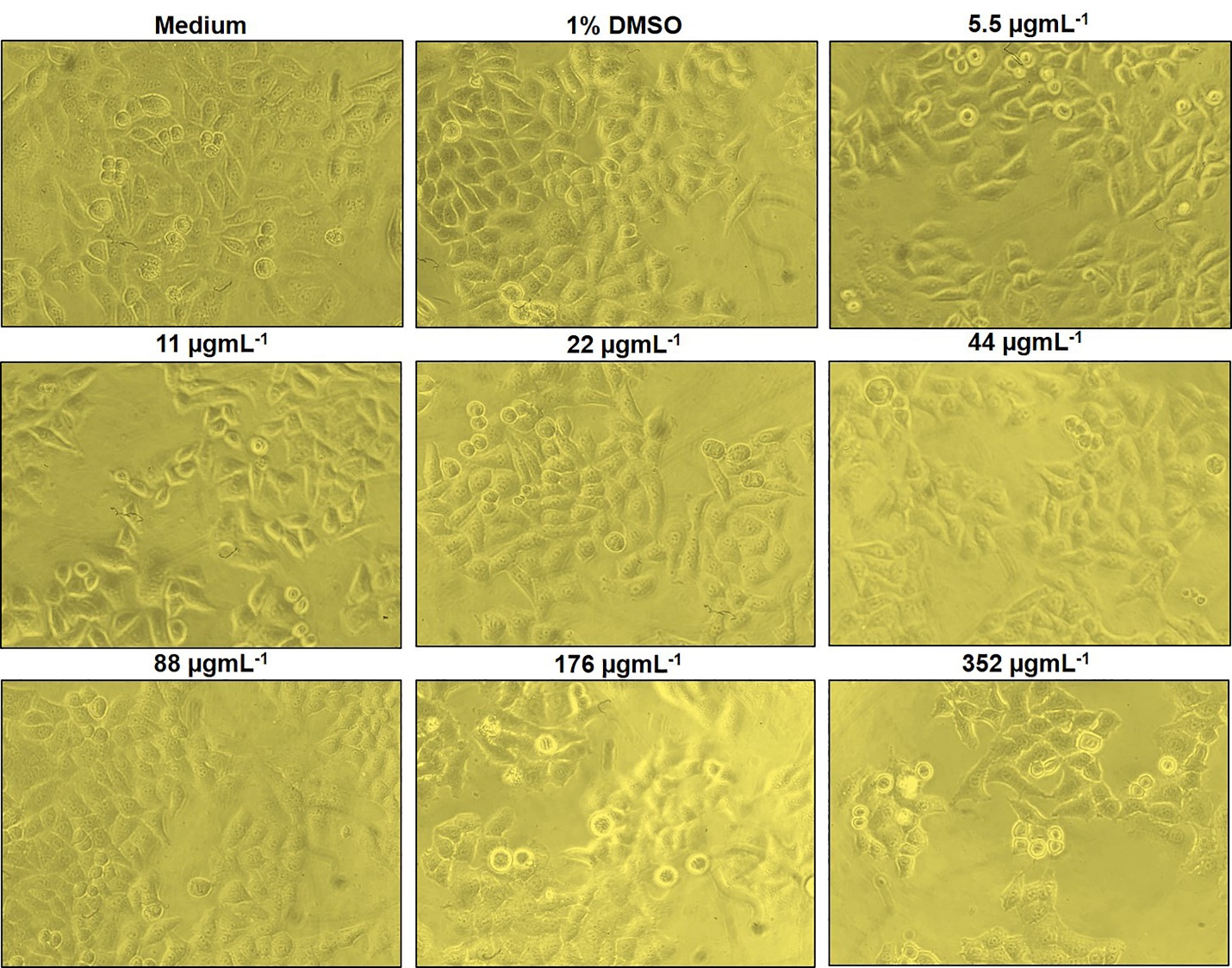


**Supplementary Figure 5.** Cytotoxic effect of antitumor peptide 3 on hepatocellular carcinoma HepG2 cells. Proliferations and morphological changes of HepG2 cells treated with different concentrations (5.5, 11, 22, 44, 88, 176, and 352 µgmL^-1^) of antitumor peptide 3, medium alone (negative control), and medium supplemented with 1% dimethyl sulfoxide (DMSO; diluent control) were observed at 24 h. They were visualized using an Olympus IX70 Inverted Tissue Culture Microscope (Olympus, Tokyo, Japan) at 200× magnification.
